# Demographics and Epidemiology of Hepatitis B in the State of Qatar: A Five-Year Surveillance-Based Incidence Study

**DOI:** 10.3390/pathogens8020068

**Published:** 2019-05-21

**Authors:** Hamad E. Al Romaihi, Nandakumar Ganesan, Elmoubasher A. Farag, Maria K. Smatti, Gheyath K. Nasrallah, Sayed M. Himatt, Moutaz F. Derbala, Maha Alshamali, Lylu K. Mahadoon, Hayat S. Khogali, Mohamed Sallam, Asmaa A. Al Thani, Mohammed Al Thani, Saad Al Kaabi, Hadi M. Yassine

**Affiliations:** 1 Ministry of Public Health, Doha 42, Qatar; halromaihi@moph.gov.qa (H.E.A.R.); nkumar@moph.gov.qa (N.G.); eabdfarag@moph.gov.qa (E.A.F.); shimatt@moph.gov.qa (S.M.H.); malshamali@moph.gov.qa (M.A.); lmahadoon@moph.gov.qa (L.K.M.); hkhogali@moph.gov.qa (H.S.K.); msallam@moph.gov.qa (M.S.); malthani@moph.gov.qa (M.A.T.); 2 Biomedical Research Center, Qatar University, Doha 2713, Qatar; msmatti@qu.edu.qa (M.K.S.); gheyath.nasrallah@qu.edu.qa (G.K.N.); aaja@qu.edu.qa (A.A.A.T.); 3 College of Health Sciences, Qatar University, QU Health, Doha 2713, Qatar; 4 Gastroenterology and Hepatology Department, Hamad Medical Corporation, Doha 3050, Qatar; mderbala@hamad.qa (M.F.D.); saadalkaabi@hamad.qa (S.A.K.)

**Keywords:** hepatitis B, surveillance, incidence, retrospective study, Qatar

## Abstract

**Background**: Expatriates represent >80% of Qatar’s population, mostly arriving from countries in Africa and Asia that are endemic with many diseases. This increases the risk for introducing new pathogens into the country and provides a platform for maintenance of endemic pathogen circulation. Here, we report on the incidence and epidemiological characteristics of hepatitis B in Qatar between 2010 and 2014. **Methods**: We performed a retrospective epidemiological data analysis using the data available at the surveillance system of the Ministry of Public Health (MOPH) in Qatar. Data were collected from distinctive public and private incorporates around the nation. Reported cases of hepatitis B patients represent those who met the stringent case definition as per World Health Organization (WHO) and Centers for Disease Control and Prevention (CDC) guidelines and eventually reported to MOPH. **Results**: The annual incidence rates of hepatitis B cases were 30.0, 34.2, 30.5, 39.4, and 19.8 per 100,000 population in 2010, 2011, 2012, 2013, and 2014, respectively. There was no specific trend or seasonality for the reported cases. The incidence rates were higher in females compared to males between 2010 and 2012, but similar in 2013 and 2014. The highest incidence rates were reported among individuals between 25 and 34 years of age. No cases were reported in children younger than five years in 2013 and 2014. Rates of hepatitis B cases declined dramatically in 2014, in both Qataris and non-Qataris, as compared to the previous years. **Conclusion**: Our results indicate a dramatic decline of hepatitis B cases in Qatar but mandate improved surveillance and vaccination efforts in expatriates in the nation.

## 1. Background

Hepatitis B virus (HBV) infection is a global public health concern. Infection with HBV impacts the liver and results in a wide range of illnesses. Individuals with chronic HBV infection are at risk of developing serious liver ailments, such as liver cirrhosis and hepatocellular carcinoma [1,2,3].

HBV can be transmitted through transfusion of infected blood, exposure to human secretions, and a needle stick or cut by a contaminated sharp object. The virus can cause both acute and chronic illnesses. Roughly, 33% of the world’s population has been exposed to the virus, and an estimated 350 million individuals are chronically infected [4,5,6,7,8]. Although hepatitis B is a common and dangerous infectious disease that can be prevented by vaccination, 887,000 individuals die every year of hepatitis B-related illnesses worldwide [9].

In the Eastern Mediterranean region, around 4.3 million individuals are infected with hepatitis B [10,11]. In the Middle East and the Indian subcontinent, WHO estimated that about 2%–5% of the population are chronically affected with hepatitis B as compared to less than 1% of the population in Western Europe and North America [12]

There is a distinct geographical variation of HBV prevalence and incidence rates in the Eastern Mediterranean region [10,13]. HBV infection rates vary from 0.6% in Iraq to higher than 8% in Sudan [14]. The prevalence of HBV in the Gulf countries is around 3.5% and 4.25%, as reported from Kuwait and Saudi Arabia, respectively [14]. In fact, a 2017 study from Saudi Arabia showed a higher rate of HBV seroprevalence reaching 7.9% [15]. Similarly, the highest incidence rate of HBV was also reported from Saudi Arabia, reaching 104.6 cases per 100,000 population [10,16]. The variation in the prevalence rates reflects the variation in the risk factors associated with the infection in each country. This highlights the need to implement effective surveillance systems on national levels to accurately monitor and control the disease spread.

Data about HBV burden and epidemiology in Qatar is scarce. Only a few earlier studies have investigated the status of HBV infection in Qatar. For instance, a 1985 report showed a 23% infection rate among acute viral hepatitis patients in Hamad General Hospital [17]. Another study conducted between 2002 and 2006 showed lower rates of HBV infection in the viral hepatitis patients reaching 4.7% [18]. However, accurate figures about the current HBV burden in Qatar are lacking.

Qatar is a hub for international travelers, and it hosts more than 1.7 million expats, most of which arrive from countries in Africa and Asia that are endemic with many diseases. Accordingly, the nation has established several programs for the detection and control of infectious diseases, including HBV [19,20]. The declining morbidity of infections in Qatar’s population can be ascribed to the rise in preventive measures that include immunization, screening of blood donations, maternal and premarital screening, and public health education and awareness. Further, the Ministry of Public Health (MOPH) has established a surveillance system to monitor the incidences of several bacterial and viral pathogens around the nation. Some of these pathogens, such as HBV, are mandatorily reported to the MOPH by the public and the private healthcare providers in Qatar. The objective of this study was to analyze the distribution and patterns of HBV infection in the State of Qatar from 2010 to 2014. The study spanned five years within the first Qatar National Health Strategy period, 2011–2016 [21]. Our study measures the trends in the epidemiology of the disease, morbidity rates by age group, sex, and nationality. 

## 2. Subjects & Methods

This is a retrospective epidemiological study based on hepatitis B surveillance data available at the MOPH, Doha, Qatar. Hepatitis B is part of officially notifiable infectious disease among 67 reportable communicable diseases in Qatar (MOPH; Hamad E. Al Romaihi’s personal communication). All primary healthcare centers around the country, including the main healthcare provider in Qatar, Hamad Medical Corporation, and all private hospitals and medical laboratories are required to notify about HBV infections to the Health Protection (HP) and Communicable Disease Center (CDC) at the MOPH. 

The HP and CDC of the MOPH use the case definition for suspected and confirmed cases of HBV according to the WHO and the CDC standards and indicators [22,23]. Briefly, in clinically suspected cases, an acute illness is defined by the distinct onset of symptoms, jaundice, or elevated serum aminotransferase levels (>2.5 times the upper limit of normal). On the other hand, a confirmed case of hepatitis B is a suspected case that is laboratory confirmed: hepatitis B surface antigen (HBsAg) positive or hepatitis B core antibody (anti-HBc-Ab) positive, and hepatitis A IgM antibody (anti-HAV-IgM) negative. Reported cases of hepatitis B represent only those infected persons who were detected, diagnosed, adhere to stringent case definition, and eventually reported to the HP and the CDC during the study period.

Data from hepatitis B cases were collected on a daily, weekly, and monthly basis by sex, age, nationality, and province. Data were initially entered into an excel sheet and then analyzed using GraphPad Prism 6 software (GraphPad Software version 6, CA, USA). Morbidity rates were calculated per 100,000 population, with the denominators used were the corresponding estimated mid-year populations compiled by Qatar Statistical Authority [24], Ministry of Development Planning and Statistics, The State of Qatar. Age groups were defined as 0–4, 5–14, 15–24, 25–34, 35–44, and ≥45 years old, and nationalities were categorized as Qatari or non-Qatari.

No ethical clearance was needed for this study as the research involved secondary data analyses without access to personal information such as names and dates of birth.

## 3. Results

### 3.1. Characteristics of the Study Group

Between 2010 and 2014, a total of 2901 hepatitis B cases were reported to the HP–CDC surveillance and outbreaks section by all sentinel sites incorporates around Qatar. The prevalence of HBV infection was standardized according to the age, gender, nationality, and date of detection to allow direct comparisons of the studied populations.

### 3.2. Yearly Distribution and Seasonality of Hepatitis B Cases

**Figure 1:**
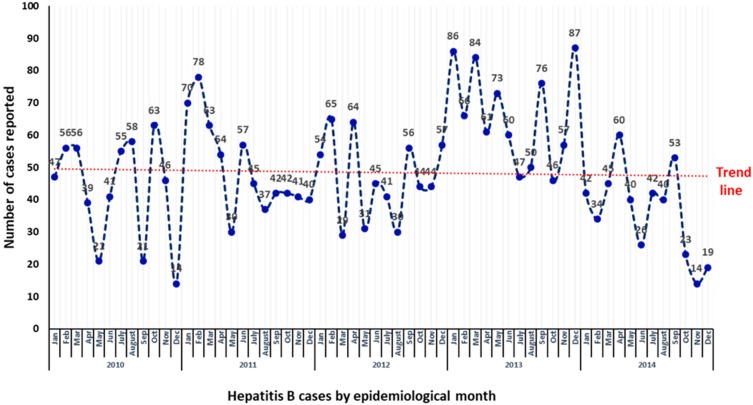
Epidemiological distribution and trends of reported hepatitis B cases in Qatar by month of notification (2010–2014).

The overall annual incidences of hepatitis B cases are summarized in Table 1. In average, 518 hepatitis B cases were reported annually in Qatar between 2010 and 2014, and they were distributed as follows: 517 (30.5 per 100,000 population) in 2010, 593 (34.2 per 100,000 population) in 2011, 560 (30.5 per 100,000 population) in 2012, 793 (39.4 per 100,000 population) in 2013, and 438 (19.8 cases per 100,000 population) in 2014. As demonstrated in Figure 1, the lowest incidence rate was reported in 2014 (*n* = 438), with a drop of 44.8% compared to the previous year (2013; *n* = 793).

**Figure 2:**
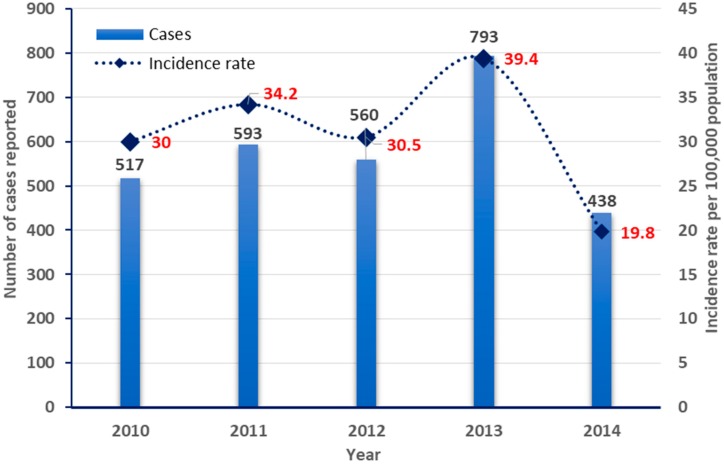
Hepatitis B: Reported cases and incidence rates by year (2010–2014).

The epidemiological distribution and trends of reported hepatitis B cases in Qatar by month of notification are depicted in Figure 2. No specific trend or seasonality of incidence cases were reported throughout the five years, where the number of cases fluctuated by the year, season, and month. There was an overall downward trend in hepatitis B cases in 2011 and 2012, followed by an increase of notified cases in the epidemiological year of 2013. A significant decline of cases was then reported in 2014 (Figure 1 and Figure 2; Table 1). 

### 3.3. Prevalence of HBV Infection by Gender, Age, and Nationality

**Figure 3:**
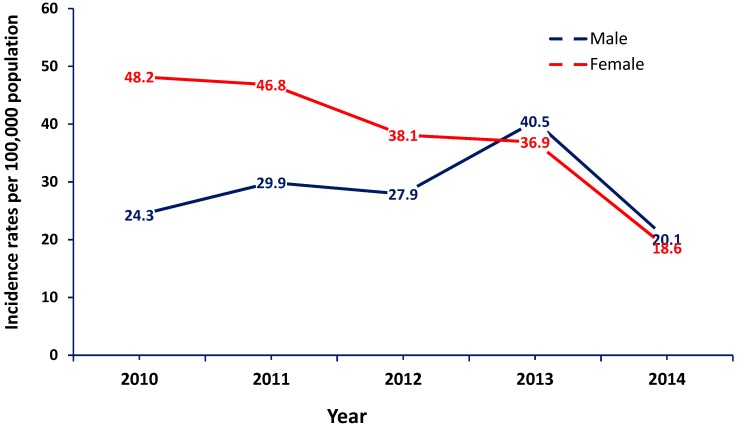
Incidence rate of hepatitis B per 100,000 population by gender (2010–2014).

Over the five-year study period, around 70% of the cases (*n* = 2010) were reported in males. Nonetheless, the overall hepatitis B incidence rate was higher among females compared to males. Interestingly, while the incidence rate was 2-fold higher in females than males in 2010, the female to male incidence rate ratio decreased gradually over the course of the study to reach around one in 2014. While the number of female cases was steady between 2012 and 2013 (38.1 and 36.9 index cases per 100,000 population), male cases increased significantly in 2013, reaching 40.5 index cases per 100,000 population. Both gender cases dropped in 2014 reaching around 20 index cases per 100,000 population (Table 1 and Table S1, and Figure 3). 

**Figure 4:**
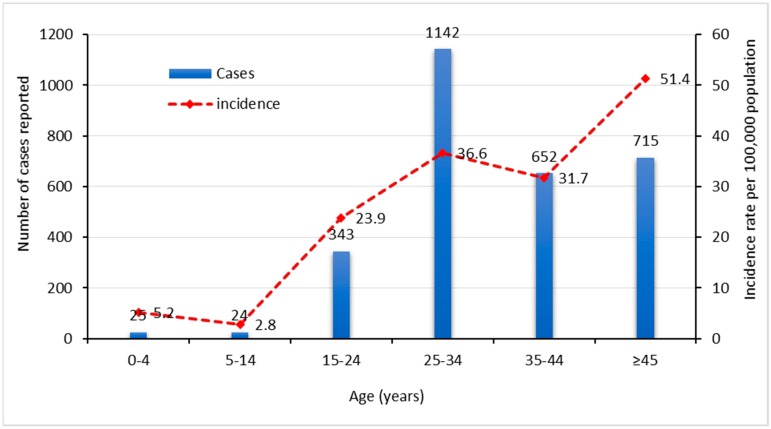
Annual reported cases and incidence rates of hepatitis B by age group (2010–2014).

Overall, most of the cases were reported in individuals between 25 and 34 years of age (*n* = 1142) (Figure 4). However, over the five-year study period, the highest incidence rate was reported in individuals ≥45 years of age, followed by the 25–34-year age group. 

**Figure 5:**
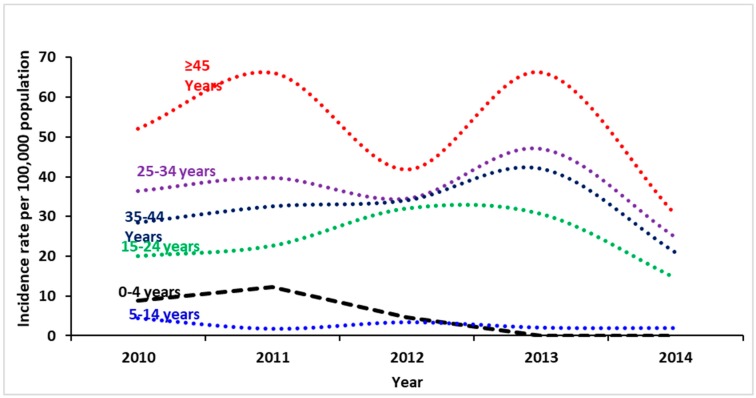
Annual specific incidence rate of hepatitis B per 100,000 population by year and age group (2010–2014).

While a substantial decrease in incidence rate was reported for patients ≥45 years in 2012, age groups of 5–14, 25–34, and 35–44 recorded a slight increase and maintained similar levels of incidence rates in the same year (32–35 cases per 100K). Minimal rates were reported for the 0–4 and 5–14 age groups over all five years, while no cases were reported in either 2013 or 2014 for the 0–4 age group. Significant decreases in the incidence rates were reported for all age groups in 2014 (Figure 4 and Figure 5 and Table S1).

In the five year period, 10.6% (*n* = 307) of the cases were Qataris compared to 2594 (89.4%) non-Qatari cases. Accordingly, reported cases of hepatitis B were much higher among the non-indigenous population with a ratio of 8.5 to 1 (Table 2).

## 4. Discussion

Similar to most of the Arabian Gulf States, Qatar hosts a significant number of expatriates constituting more than 80% of its residents, most of which arrive from countries in Asia and Africa suffering from weak healthcare systems and, thus, are endemic with many diseases. Despite the Medical Commission of the MOPH test for HBV in all expatriates arriving in Doha [20], the actual burden and epidemiology of this virus in the nation have not been well studied. Fortunately, Qatar has an excellent healthcare system, which has been ranked 13th best in the world and first in the Middle East by the 2017 Legatum Prosperity Index [25]. In part, this system involves the identification and registration of infectious diseases at the MOPH. Such data are regularly reported to MOPH from all healthcare providers around Qatar. Accordingly, Qatar national surveillance data provide estimates of the patterns, trends, and burdens of infectious diseases in the community, and help in developing control and prevention strategies such as the implementation of effective vaccination programs. 

HBV is one of the most important causes of chronic hepatitis, cirrhosis, and hepatocellular carcinoma, and it is an all-pervading infection with a worldwide distribution [26,27]. Despite the few studies on the hepatitis B situation in the Arabic world, the prevalence of chronic HBV infection was found to be decreasing in some Arab countries, such as Arabian Gulf region, Lebanon, Egypt, and Libya [28]. This could be partially attributed to the expanded immunization programs in these nations. Among the Arabian Gulf States, HBV burden has been well studied in the Kingdom of Saudi Arabia (KSA), where its prevalence and serostatus has been determined for specific subpopulations. According to the 2010 figures, HBV prevalence in KSA in the general population ranged from 1.5% to 2.6%, whereas some other reports describe a prevalence rate of >8% [28]. Similar rates, ranging between 2% and 7%, were also reported in the United Arab Emirates. In Qatar, regardless of regular testing for HBV at the Medical Commission for all expatriates entering the country, as well as all suspected cases in hospitals and primary healthcare centers, the actual figure for hepatitis B virus status is lacking. Here, we provide the first report on the epidemiology of hepatitis B in Qatar between 2010 and 2014. The study spanned five years representing the period of the first Qatar National Health Strategy, 2011–2016.

During the first three years of the study (2010–2012), we reported an equivalent number of cases averaging at 556 cases per year. Interestingly, we recorded a noticeable increase in the number of cases in 2013 (*n* = 793), which was then significantly dropped in 2014 (*n* = 438), recording the lowest number during the study period. The increase in 2013 cases could be partially attributed to the inflow of construction workers that entered the country in that period. On average, the incidence rate of hepatitis B was around 31 per 100,000, which is significantly lower than what has been reported in the neighboring countries [10]. Expectedly, most of the cases throughout the five years were reported in non-Qataris (89.4%), which represent more than 80% of the population. Similarly, most of the infections occurred in males (average of 69.3%), where the percentage of infected males gradually increased from 61.9% in 2010 to 76.0% in 2014, presumably due to the continuous demand of male workers in the construction sector. Nonetheless, the incidence rate of hepatitis B was higher in females compared to male, due to the dominant male population in the nation, most of whom are living alone in the industrial sites [24]. In fact, male dominance in HBV infections has been observed by multiple previous reports [29]. It was shown that HBV infection affects males four times more than females, suggesting the possible role of the estrogen hormone in the protection and defense against HBV infection [29].

The factor of male expatriates seems to also impose an effect on the distribution of the disease among different age groups, where the highest number of cases (*n* = 1142) were reported in individuals between 25 and 34 years of age. All the above observations suggest the contribution of expatriates in the dissemination of the disease, which mandates specific regulatory actions targeting this group, whether by immunization or other preventive methods. On the other hand, very low numbers of HBV infections were reported in individuals younger than 14 years, reaching zero in the 0–4-year age group in 2013 and was maintained in 2014. Similar results were obtained in reports from Canada, Iran, and China [30,31,32]. It has been shown that lower HBV infection rates were detected among young age groups, whereas individuals between 25 and 50 years old were the most affected age group. This observation is partially attributed to the early vaccination strategy implemented in many countries, including Qatar, where the HBV vaccine is provided free of charge for all newly born children in Qatar [33]. A 2018 study on HBV immunization among school students in Qatar showed that all Qatari children were vaccinated, and none of the tested students (Qataris and non-Qataris) had HBV infection [34]. 

Qatar is already on the path toward eliminating viral hepatitis, and this is noted by the dramatic drop in the number of cases in 2014. Every newcomer to Qatar must undergo the tests at the Medical Commission to detect contagious diseases including hepatitis B and hepatitis C. Other strategies to reduce the prevalence and burden of hepatitis in Qatar include the screening of all pregnant women, blood donors, and healthcare workers for the virus. Furthermore, screening for the virus is done for certain professions (e.g., barbers) as part of the licensing process. MOPH also provides vaccination free of charge to those who are not immune to the virus [35]. Importantly, Qatar has established a new Communicable Diseases Center (CDC Qatar) which receive referrals from the Primary Health Care Corporation (PHCC) and private sectors of people infected with hepatitis for critical care management, to minimize the risk of virus transmission in the community.

Considering this is the first study of HBV status in Qatar, the study suffers from several limitations: The study reports on the identified cases at hospitals and primary healthcare centers only, infection status has not been determined among specific subpopulations, factors linked to HBV transmission have not been determined, specific nationalities and length of stay in Qatar have not been recorded, and vaccination rates have not been determined. Nonetheless, this study could be a cornerstone for future studies that look at the epidemiological factors associated with the HBV infections in the general population, as well as specific subpopulations like healthcare workers and blood donors. In addition to young children, immunization is provided freely for seronegative individuals, but it is done passively for those who seek medical attention. Hence, immunization campaigns like those organized for influenza and measles, mumps, and rubella (MMR) should be implemented for HBV. With the vision of making the State of Qatar free of viral hepatitis, awareness activities, increased diagnosis, and key interventions are needed.

## 5. Conclusions

Regardless of the regular testing for HBV at Medical Commission for all expatriates entering the country, as well as all suspected cases in hospitals and primary healthcare centers, the actual figure for hepatitis B virus status is lacking. Here, we provide the first report on the epidemiology of hepatitis B in Qatar between 2010 and 2014. Considering that most of the cases throughout the five years were reported in non-Qataris (89.4%), which represent more than 80% of the population, regulatory actions targeting this group, whether by immunization or other preventive methods are required. Nonetheless, the continuous decreases of the number of cases in the past years reflect the efficiency of the control program implemented by the MOPH, including the vaccination of all newborn babies and screening for the virus in all expatriates that enter the country. Further followup studies are needed in the future. 

## Figures and Tables

**Table 1:** Distribution of reported hepatitis B by gender between 2010 and 2014.

Epidemiological Year	Gender		Total N (%)
	Male N (%)	Female N (%)	
2010	315 (61.9%)	202 (39.1%)	517 (100%)
2011	385 (64.9%)	208 (35.1)	593 (100%)
2012	378 (67.5%)	182 (32.5)	560 (100%)
2013	599 (75.5%)	194 (24.5%)	793 (100%)
2014	333 (76.0%)	105 (24.0%)	438 (100%)
Overall	2010 (69.3%)	891 (30.7%)	2901 (100%)

**Table 2:** Distribution of reported hepatitis B cases by nationality between 2010 and 2014.

Epidemiological Year	Nationality		Total N (%)
	Qatari N (%)	Non-Qatari N (%)	
2010	81 (15.7)	436 (84.3)	517 (100%)
2011	76 (12.8)	517 (87.2)	593 (100%)
2012	62 (11.7)	498 (88.9)	560 (100%)
2013	61 (7.7)	732 (92.3)	793 (100%)
2014	27 (6.2)	411 (93.8)	438 (100%)
Overall	307 (10.6%)	2594 (89.4%)	2901 (100%)

## Supplementary Materials

The following are available online at https://www.mdpi.com/2076-0817/8/2/68/s1, Table S1: Distribution of hepatitis B reported cases and incidence rates among different groups (2010–2014).
