# Supplementary material for: Demographics and Epidemiology of Hepatitis B in the State of Qatar: A Five-Year Surveillance-Based Incidence Study
Source: Pathogens. 2019 May 21;8(2):68. doi: 10.3390/pathogens8020068 (PMC6630982; doi:10.3390/pathogens8020068)
Supplement: Supplementary file 1 [file pathogens-08-00068-s001.pdf]

Table S1. Distribution of hepatitis B reported cases and incidence rates among different groups (2010–2014).

	Epidemiological year									
	**2010**		**2011**		**2012**		**2013**		**2014**	
	**Cases**	**Incidence rate**	**Cases**	**Incidence rate**	**Cases**	**Incidence rate**	**Cases**	**Incidence rate**	**Cases**	**Incidence rate**
Gender										
Male	315	24.3	385	29.9	378	27.9	599	40.5	333	20.1
Female	202	48.2	208	46.8	182	38.1	194	36.9	105	18.6
Age										
0–4	8	8.8	12	12.2	5	4.7	0	0	0	0
5–14	7	4.5	3	1.8	6	3.5	4	2.1	4	2
15–24	53	20	62	22.6	90	32	90	30.6	48	14.4
25–34	199	36.5	229	39.8	211	34.6	311	47.1	184	24.8
35–44	118	28.5	118	32.6	133	34.1	191	42.1	102	20.9
≥45	132	52.1	169	66.2	115	41.8	197	66.3	100	30.3
Overall	517	30	593	34.2	560	30.5	793	39.4	438	19.8
